# Validation of multispectral imaging–based tissue oxygen saturation detecting system for wound healing recognition on open wounds

**DOI:** 10.1117/1.JBO.29.8.086004

**Published:** 2024-08-13

**Authors:** Yi-Syuan Shin, Kuo-Shu Hung, Chung-Te Tsai, Meng-Hsuan Wu, Chih-Lung Lin, Yuan-Yu Hsueh

**Affiliations:** a National Cheng Kung University, College of Medicine, National Cheng Kung University Hospital, Department of Surgery, Division of Plastic and Reconstructive Surgery, Tainan, Taiwan; b National Cheng Kung University, College of Medicine, Institute of Clinical Medicine, Tainan, Taiwan; c National Cheng Kung University, Department of Electrical Engineering, Tainan, Taiwan; d National Cheng Kung University, College of Medicine, Department of Physiology, Tainan, Taiwan

**Keywords:** tissue oxygen saturation, multispectral imaging-based, wound healing, proliferative phase, angiogenesis

## Abstract

**Significance:**

The multispectral imaging–based tissue oxygen saturation detecting (TOSD) system offers deeper penetration (∼2 to 3 mm) and comprehensive tissue oxygen saturation (${\mathrm{StO}}_{2}$) assessment and recognizes the wound healing phase at a low cost and computational requirement. The potential for miniaturization and integration of TOSD into telemedicine platforms could revolutionize wound care in the challenging pandemic era.

**Aim:**

We aim to validate TOSD’s application in detecting ${\mathrm{StO}}_{2}$ by comparing it with wound closure rates and laser speckle contrast imaging (LSCI), demonstrating TOSD’s ability to recognize the wound healing process.

**Approach:**

Utilizing a murine model, we compared TOSD with digital photography and LSCI for comprehensive wound observation in five mice with 6-mm back wounds. Sequential biochemical analysis of wound discharge was investigated for the translational relevance of TOSD.

**Results:**

TOSD demonstrated constant signals on unwounded skin with differential changes on open wounds. Compared with LSCI, TOSD provides indicative recognition of the proliferative phase during wound healing, with a higher correlation coefficient to wound closure rate (TOSD: 0.58; LSCI: 0.44). ${\mathrm{StO}}_{2}$ detected by TOSD was further correlated with proliferative phase angiogenesis markers.

**Conclusions:**

Our findings suggest TOSD’s enhanced utility in wound management protocols, evaluating clinical staging and therapeutic outcomes. By offering a noncontact, convenient monitoring tool, TOSD can be applied to telemedicine, aiming to advance wound care and regeneration, potentially improving patient outcomes and reducing healthcare costs associated with chronic wounds.

## 1 Introduction

Annual costs for wound care in the United States ranged from $28.1 billion to $31.7 billion.^1^ In the context of problematic cases, hospital-based wound clinics had reported wound healing rates as low as 30.5%.^2^ Wound healing is a dynamic and complex process, encompassing four phases: the hemostatic phase, the inflammatory phase, the proliferative phase, and the remodeling phase.^3^ Among these phases, the proliferative phase is recognized as the most crucial stage for wound healing, attracting extensive regenerative approaches.^4^ Tissue oxygenation is considered the key factor during the proliferative phase, and local tissue hypoxia is widely accepted as an indicator of impaired wound healing.^5^ Therefore, effective monitoring of wound condition necessitates methods for assessing wound tissue oxygenation, highlighting its importance as a key target especially in open wounds.

Various methods have been proposed for assessing wound tissue oxygenation, encompassing both direct and indirect, as well as invasive and noninvasive approaches.^6^ Although invasive assessments have disadvantages such as increasing the risk of infection, additional pain and discomfort for patients, and trauma to the wound site, which could delay wound healing, noninvasive wound assessments are preferred. Noninvasive approaches include laser speckle contrast imaging (LSCI), hyperspectral imaging (HSI), and multispectral imaging (MSI). The principle of LSCI is to monitor the movement of red blood cells within cutaneous tissues. By calculating the changes in the frequency and speckling of the reflected laser light captured, blood perfusion and flux can be estimated, respectively.^7^^–^^9^ However, the expense of the procedures is a lot, and their susceptibility to interference by ambient light limits the clinical application of LSCI.^10^

In comparison, under environments affected by ambient light, HSI and MSI can generate reliable results.^11^^–^^14^ HSI emits broadband light within visible and near-infrared (NIR) wavelengths and thus can acquire oxygenation parameters of cutaneous tissue, such as oxyhemoglobin (${\mathrm{HbO}}_{2}$), deoxyhemoglobin (Hb), and tissue oxygen saturation (${\mathrm{StO}}_{2}$). However, HSI has the disadvantage of complex calculation processes and high computational power. MSI, a simplified version of HSI, captures a few specific bands covering the spectrum from the visible to NIR. MSI yields the same parameters as HSI using simple hardware structures.^6^^,^^15^ Overall, MSI has a lower cost and requires less computational power than LSCI and HSI.^16^

Systems utilizing NIR wavelength exhibit substantial potential due to their capacity for deep tissue penetration (millimeters to centimeters).^17^ Time-domain near-infrared spectroscopy (TD-NIRS) is another technique that uses NIR light for determining ${\mathrm{StO}}_{2}$, with a particular focus on probing internal tissues such as the brain using measurements conducted on the head.^18^ TD-NIRS requires an advanced system capable of measuring both the wavelength and the arrival time of single photons.^19^ This information allows the determination of ${\mathrm{StO}}_{2}$ at different depths, in both superficial and deeper tissues.^20^ NIR light passes harmlessly through the skin and possesses minimal tissue absorption and preferential scattering by hemoglobin.^21^ Hemoglobin undergoes spectroscopic and magnetic property changes corresponding to its degree of oxygenation, with ${\mathrm{HbO}}_{2}$ and Hb exhibiting different absorption spectra in the NIR range.^19^ The discernible differences in reflected light can thus be employed to detect physiological changes indicative of tissue oxygenation. Due to the significant interest in tissue oxygenation measurement, NIR imaging has been applied in prior research on conditions such as diabetic ulcers, decubitus ulcers, burn wounds, and chronic wounds.^22^^–^^28^ However, in related studies, none have validated NIR imaging concurrently with other imaging systems and biochemical methods.

Our study introduced an MSI system based on NIR for detecting ${\mathrm{StO}}_{2}$, which we refer to as the tissue oxygen saturation detecting (TOSD) system. As TOSD is a relatively new technique, it is crucial to cross-validate it and compare its performance with established techniques that also contribute to evaluating wound conditions, such as LSCI.^7^^,^^9^ We hypothesize that the TOSD system will be more sensitive and accurate in correlating to ${\mathrm{StO}}_{2}$ compared with LSCI due to its use of NIR wavelengths, which allow for deeper tissue penetration and minimal interference from ambient light. In addition, the TOSD system’s measurement of ${\mathrm{StO}}_{2}$ based on the absorption spectra of ${\mathrm{HbO}}_{2}$ and Hb may provide a more reliable assessment compared with the implicit relationship of ${\mathrm{StO}}_{2}$ with blood flow measured by LSCI. To validate the performance of the TOSD system, we employed various methods in an animal model platform. Three imaging systems, namely, digital photography, TOSD, and LSCI, were used to detect tissue oxygenation in open wounds. Furthermore, we analyzed the biochemistry profile of wound discharge to provide additional support for the translational significance of the TOSD system.

## 2 Materials and Methods

### 2.1 Animal Wound Model

**Fig. 1:**
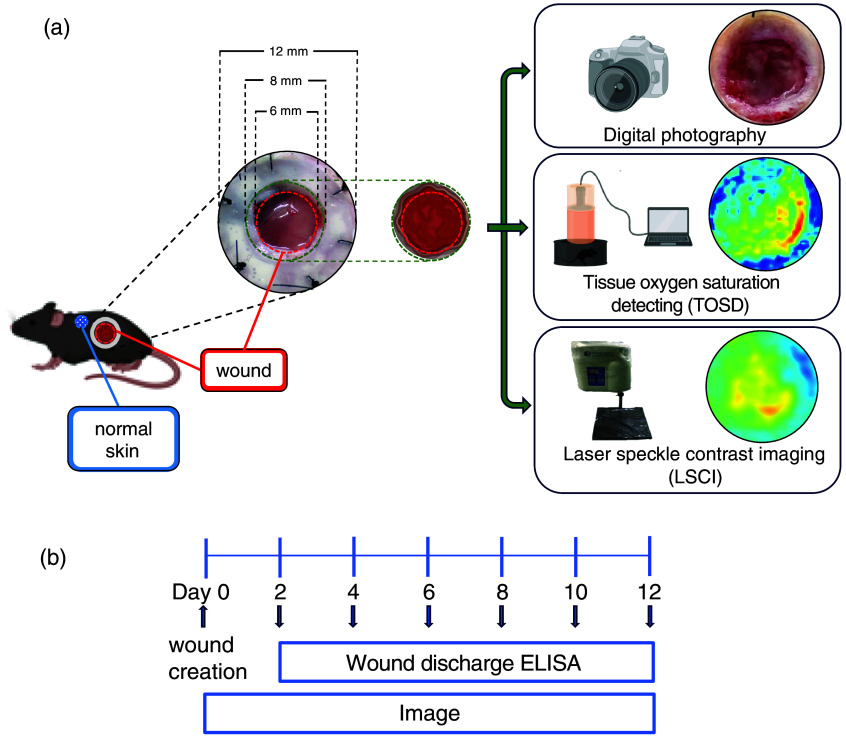
Schematic summary of this research. (a) Following the Galiano method, a 6-mm full-thickness excisional wound model with silicon ring splinting was created over the back unilaterally, and the wound was recorded by digital photography, tissue oxygen saturation detecting, and laser speckle contrast imaging. (b) Timeline of the experimental overview of open wounding healing and image recording.

Mice aged 6 to 8 weeks were housed at the National Cheng Kung University Laboratory Animal Center, under a regulated environment. All mice were maintained in rooms with a temperature range of 18 to 26°C, relative humidity of 30% to 70%, and a regular light/dark cycle of 12/12 h to fit their circadian rhythm. The air quality in the animal rooms was maintained using a high-efficiency particulate air filter. The experiment was conducted under the supervision of the Institutional Animal Care and Use Committee (IACUC approval numbers 111273 and 112060). A 6-mm splinted full-thickness excisional wound model was generated following the established methods of Galiano et al.^29^ One full-thickness dermal wound, measuring 6 mm in diameter, was surgically created on the left side of the dorsum [Fig. 1(a)]. To prevent contraction of the wound by the panniculus carnosus muscle, a silicone ring was employed, and the wound was secured with eight interrupted sutures using 6-0 nylon. Tegaderm was applied to cover the wounds after construction, and the dressings were changed every other day. Photographs of the wounds were captured using three imaging systems at the time of surgery and during each dressing change over a 12-day period. Wounding was initiated on day 0, with a subsequent observation of wound imaging and discharge sampling on each experimental day [Fig. 1(b)].

### 2.2 Imaging System

Based on the mouse excisional wound model described previously, the created wound was recorded by three imaging systems: digital photography (Stylus TG3 Tough, Olympus, Tokyo, Japan), TOSD, and LSCI [Fig. 1(a)]. We have already established the TOSD system to measure ${\mathrm{StO}}_{2}$.^16^ Detailed illustrations of the theory of cutaneous tissue optics and the TOSD setup are described in our collaborating work. TOSD is an MSI-based tissue oxygenation measurement system that provides an indirect method of tissue oxygenation measuring by irradiating the tissue with two wavelengths of red light (660 nm) and NIR (880 nm). Both lights penetrate the superficial tissue of the skin layer, and the reflected and scattered lights captured by a monochrome camera were utilized to calculate the ${\mathrm{StO}}_{2}$ levels.^30^ The TOSD system consists of a monochrome camera (MB152USB, OMRON SENTECH, Ebina, Japan), a light-emitting diode (LED) light module, and a control unit for synchronizing the camera and LEDs. Under the irradiation of the LED light module, the reflected ($I_{1}$) and scattered ($I_{2}$) lights from the tissue are captured by the monochrome camera. Hemoglobin with different degrees of oxygenation contributes to considerable differences in the reflected images. Oxygenation parameters, including ${\mathrm{HbO}}_{2}$, Hb, and ${\mathrm{StO}}_{2}$, can be computed through the captured images.

Following the steps shown below, estimating ${\mathrm{StO}}_{2}$ levels was calculated^31^ [Fig. S1 in the Supplementary Material]. The modified Beer–Lambert law describing the relationship between light and chromophores is used in Eq. (1),^32^ where λ is the wavelength of the light source, OD(λ) is the optical density, which equals the sum of the absorbances in cutaneous tissues, $I_{0}$ is the intensity of incident light, $I_{1}$ is the intensity of reflected light, and $I_{2}$ is the intensity of the scattered light. ε is the absorption coefficient of chromophores, and C is the concentration of chromophores. d(λ) is the maximum depth of penetration of the light (λ) in cutaneous tissues

$$\text{Modified Beer}-\text{Lambert law}:\;\mathrm{OD}(\lambda )=\ln\frac{I_{0}-I_{1}}{I_{2}-I_{1}}=\ln\;\frac{I_{0}-R*I_{0}}{I_{2}-R*I_{0}}\;=(\varepsilon_{\mathrm{Hb}}c_{\mathrm{Hb}}+\varepsilon_{{\mathrm{HbO}}_{2}}c_{{\mathrm{HbO}}_{2}}+\varepsilon_{\text{water}}c_{\text{water}}+\varepsilon_{\text{other}}c_{\text{other}})*d(\lambda ).$$ (1)

By calculating the intensity of light captured by the TOSD system, the values of ${\mathrm{OD}}_{660}$ and ${\mathrm{OD}}_{880}$ are obtained. Although the other values are constants or calculated, the unknown concentration of chromophores ($C_{\mathrm{Hb}}$ and $C_{{\mathrm{HbO}}_{2}}$) therefore can be deducted. Values of ${\mathrm{StO}}_{2}$, representing the levels of cutaneous tissues, can be derived using Eq. (3)

$$\text{Using }\lambda_{1}=660\;\mathrm{nm},\;\lambda_{2}=880\;\mathrm{nm}\text{ to solve }C_{\mathrm{Hb}},C_{{\mathrm{HbO}}_{2}}\;\frac{\varepsilon_{\mathrm{Hb}}^{\lambda_{1}}c_{\mathrm{Hb}}+\varepsilon_{{\mathrm{HbO}}_{2}}^{\lambda_{1}}c_{{\mathrm{HbO}}_{2}}}{\varepsilon_{\mathrm{Hb}}^{\lambda_{2}}c_{\mathrm{Hb}}+\varepsilon_{{\mathrm{HbO}}_{2}}^{\lambda_{2}}c_{{\mathrm{HbO}}_{2}}}=\frac{\frac{\mathrm{OD}(\lambda_{1})}{d(\lambda_{1})}-\varepsilon_{w}^{\lambda_{1}}c_{w}-\varepsilon_{o}^{\lambda_{1}}c_{o}}{\frac{\mathrm{OD}(\lambda_{2})}{d(\lambda_{2})}-\varepsilon_{w}^{\lambda_{2}}c_{w}-\varepsilon_{o}^{\lambda_{2}}c_{o}\;},$$ (2)

$$\text{Derived calculated value of }{\mathrm{StO}}_{2}=\frac{C_{{\mathrm{HbO}}_{2}}}{C_{\mathrm{Hb}}+C_{{\mathrm{HbO}}_{2}}}.$$ (3)

In our study, LSCI (moorFLPI-2, Moor Instruments, Devon, United Kingdom) was applied to validate the TOSD system. The LSCI technique quantifies the blurring of the speckle pattern caused by the motion of scattering particles (i.e., red blood cells) by calculating the speckle contrast. Speckle patterns are created based on the interactions between photons and moving red blood cells, which cause Doppler shifts in the frequency of the scattered light.^7^^,^^8^^,^^10^ LSCI captures the dynamic speckle patterns created, and the contrast of the speckle pattern is inversely related to the blood flow in the tissue.^7^^,^^8^^,^^10^ The LSCI system used in this study illuminates the tissue using infrared laser diodes with a center wavelength of 785 nm. The camera is positioned at a fixed distance of 25 cm from the measured object using two aiming lasers. By analyzing the captured speckle patterns based on the interaction between photons and moving red blood cells, a 16-color–coded tissue perfusion image is then created.^9^

During each experimental day, TOSD and LSCI were used simultaneously to obtain information on the open wound through our animal model platform. The images captured by TOSD and LSCI were analyzed to assess the flare intensity within the regions of interest (ROIs). The mean values within those ROIs (i.e., wound and normal skin) were computed. The ROIs were provided by two experienced plastic surgeons at the National Cheng Kung University Hospital in Tainan, Taiwan.

### 2.3 Wound Area Analysis

The wound area was documented on alternate days throughout a 12-day experimental period. Each experimental day involved capturing photographs using three distinct imaging systems. Wound measurements were conducted using the freehand tool in LabelMe (ver. 4.6.0), a project developed by the MIT Computer Science and Artificial Intelligence Laboratory, offering a dataset of digital images with annotations. The determination of wound edges was independently performed by two observers within our team, adhering to a blinded protocol. It was ensured that the team of observers was composed of two independent senior surgeons to enhance accuracy. The wound closure rate was computed using the following equation:

$$\text{wound closure rate}=\frac{\text{wound area on day }n-\text{wound area on day }m}{m-n}.$$ (4)

### 2.4 Enzyme-Linked Immunosorbent Assay (ELISA)

To assess the presence of angiogenic proteins in wound discharge, an analysis was conducted using a RayBio^®^ C-Series Mouse Angiogenic Protein Antibody Array (RayBiotech Inc., Norcross, Georgia, United States). Wound discharge samples were collected at each dressing change over a 12-day period. The RayBiotech Mouse Angiogenesis Kit (Catalog No. AAM-ANG-1-2) was employed for ELISA. The experimental protocol was strictly adhered to as per the provided instructions. Following proper lysis and homogenization of wound discharge, the protein concentration of lysates was determined. Subsequently, 50 to 500  μg of total protein was loaded on each membrane. Quantification of protein expression levels was performed by assessing the color intensity of individual spots on the membranes relative to the positive control spots prebound to the membranes. The final results are presented as mean ± SD, derived from independent experiments.

### 2.5 Statistical Methods

Statistical analyses were performed using Prism (ver. 9.0, GraphPad, San Diego, California, United States). All continuous data are presented as mean ± standard deviation. One-way analysis of variance (ANOVA) with Tukey’s multiple comparison test and Sidak’s multiple comparison test were employed for datasets, including quantification of the wound area over time, quantification of ${\mathrm{StO}}_{2}$, and blood flux in normal skin as well as wound during the experiment period and levels of protein expressions on different days. Comparisons of ROIs between wounds and normal skin on each day were performed using two-way ANOVA. Statistical significance was defined at a threshold of p<0.05.

## 3 Results

### 3.1 Wound Closure Rate Accelerates During Proliferative Phase in Mouse Excisional Wound Model

**Fig. 2:**
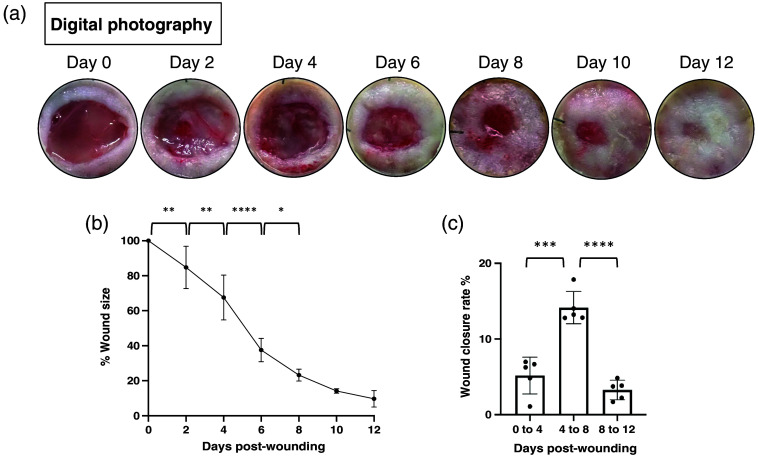
Excisional wound healing from days 0 to 12. (a) Representative image of the wound taken by digital photography. (b), (c) Quantification of the wound area over time. Data are presented as mean value ± SD (n=5). *p<0.05 by one-way ANOVA.

A full-thickness wound was created on the back of each mouse and splinted by a silicon ring to generate an excisional wound model on day 0. Digital photographs taken during each dressing change revealed the trend of wound healing throughout the experimental period [Fig. 2(a)]. We further quantified the changes in wound area and wound closure rate over time [Fig. 2(b), Fig. S2 in the Supplementary Material]. The size of the open wound reduced progressively, showing a significant difference from days 2 to 8. Wound closure rates were calculated and compared within three phases: days 0 to 4, days 4 to 8, and days 8 to 12. Notably, during the middle phase (days 4 to 8), wounds exhibited a greater closure percentage compared with the early and late phases of wound healing [Fig. 2(c)].

### 3.2 TOSD and LSCI Both Reveal Identical Signal Changes During Wound Healing Process

**Fig. 3:**
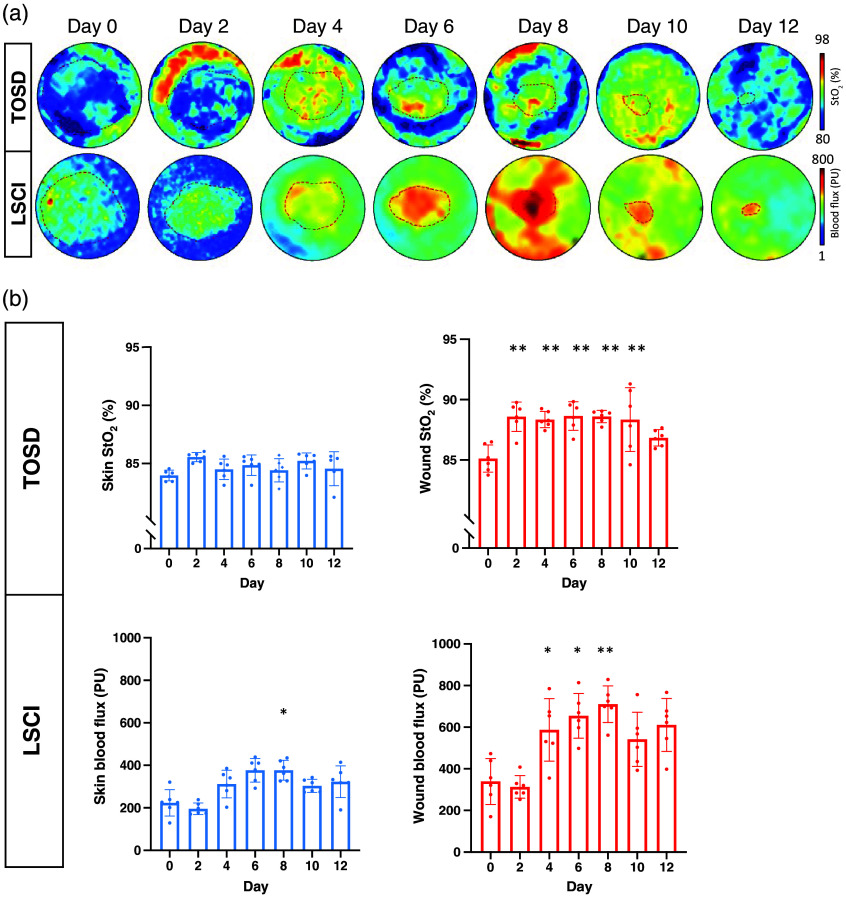
Sequential image recording during wound healing from days 0 to 12. In TOSD, ${\mathrm{StO}}_{2}$ was measured in percentage (%), whereas blood flux was measured by LSCI in perfusion units (PU). (a) Upper panel: tissue oxygenation can be indicated by tissue microcirculation detected via TOSD. Lower panel: perfusion measured by LSCI. The wound region is circled by a red dashed line. (b) Quantification of TOSD and LSCI between normal skin (blue color, left column) and open wound (red color, right column). Data are presented as mean value ± SD (n=5). *p<0.05 by one-way ANOVA.

Throughout the wound healing phase, we conducted serial monitoring of the open wound for 12 days after wound creation, comparing the results obtained by both TOSD and LSCI. Initially, we established that the measured mean and median ${\mathrm{StO}}_{2}$ values were not statistically different for both the open wound and the unwounded skin (Fig. S3 in the Supplementary Material). Consequently, the measured mean ${\mathrm{StO}}_{2}$ values were used in the subsequent analysis. Representative images indicated that tissue oxygenation levels measured by TOSD exhibited similar trends of change when compared with tissue blood flow measured by LSCI [Fig. 3(a)]. In addition, stable measured signals were observed for the unwounded skin using both TOSD and LSCI [Fig. 3(b)]. In the open wound, TOSD detected a significant increase in ${\mathrm{StO}}_{2}$ from days 2 to 10, compared with day 0. LSCI demonstrated an identical trend of signal change during the middle phase of wound healing [Fig. 3(b)].

### 3.3 TOSD and LSCI Detect Significant Signal Increase of Open Wound as Compared with Unwounded Skin

**Fig. 4:**
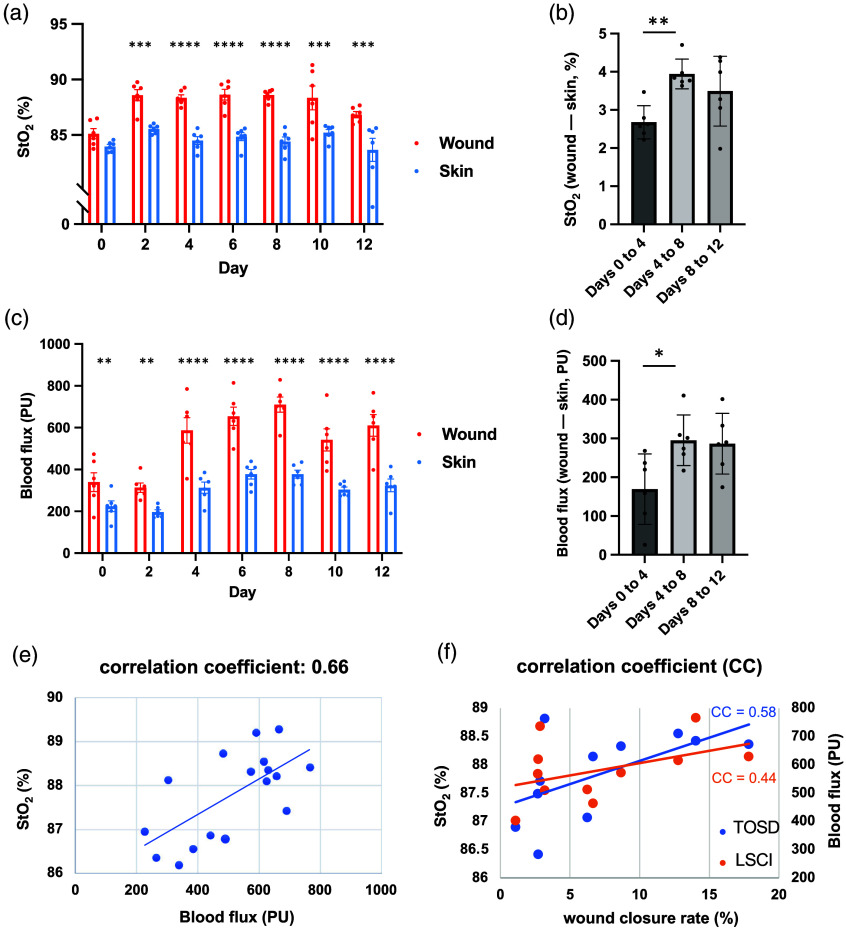
Trend of detected signals by TOSD and LSCI during wound healing. ${\mathrm{StO}}_{2}$ was measured by TOSD in percentage (%), whereas blood flux was measured by LSCI in perfusion units. (a), (b) Tissue oxygenation by TOSD among wound (red color) and unwound normal skin (blue color). (c), (d) Flux parameter measured by LSCI. Differences in tissue oxygenation between unwounded skin and wound in different phases were calculated and are shown in panels (b) and (d), using TOSD and LSCI, respectively. (e) Correlation coefficient between TOSD and LSCI. (f) Correlation coefficient between wound closure rate and two imaging systems. The linear correlation between wound closure rate and TOSD is shown in blue with a coefficient of 0.58, whereas the linear correlation between wound closure rate and LSCI is shown in orange with a coefficient of 0.44. Data are presented as mean value ± SD (n=5). *p<0.05, **p<0.01, ***p<0.001, and ****p<0.0001 by one-way ANOVA.

To further validate the significance of the detected ${\mathrm{StO}}_{2}$ signals by TOSD, we compared the values of ${\mathrm{StO}}_{2}$ between the unwounded skin and the wound on each experimental day. The detected ${\mathrm{StO}}_{2}$ of the open wound revealed a significant increase to that of the unwound skin from days 2 to 12 [Fig. 4(a)]. Moreover, such differences between the wound and the skin had mostly significant distinctions in days 4 to 8, the middle phase of wound healing [Fig. 4(b)]. The serial signals from LSCI also demonstrated a similar trend [Figs. 4(c) and 4(d)]. Given that TOSD and LSCI exhibited similar performance, we calculated a similarity measurement. A high correlation was found, and the correlation coefficient between these two systems is 0.66 [Fig. 4(e)]. We further analyzed the accuracy of these two systems by direct comparison with wound closure rate. The correlation coefficient of TOSD with wound closure rate is 0.58, which is higher than that of LSCI, which is 0.44 [Fig. 4(f)].

### 3.4 Increased Expression of Angiogenesis Markers During Proliferative Phase Correlated with Wound Tissue Oxygenation

**Fig. 5:**
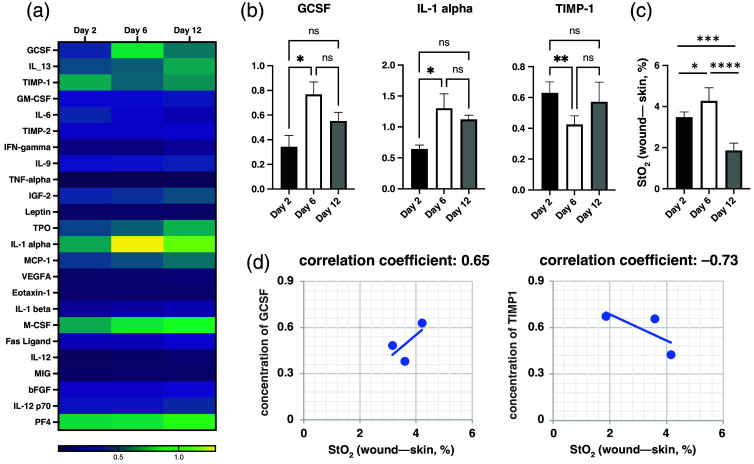
Biochemical analysis of secreted protein during wound healing. (a) Heatmap of proteins relating to mouse angiogenesis. Signature cytokines in different phases during wound healing are revealed. (b) Protein expression of GCSF, IL-1α, and TIMP-1. (c) Differences between tissue oxygenation of wounds and unwounded skin during post-wounding days 0 to 4, 4 to 8, and 8 to 12 were measured via TOSD. (d) Correlation coefficient of tissue oxygenation to GCSF (0.65, left) and TIMP-1 (−0.73, right). Data are presented as mean value ± SD (n=5). *p<0.05, **p<0.01, ***p<0.001, and ****p<0.0001 by one-way ANOVA.

To further assess the significant signal changes detected during the middle phase of wound healing by TOSD, wound fluid discharge was collected and analyzed using an angiogenesis ELISA kit [Fig. 5(a)]. Signature cytokines of the proliferative phase during wound healing, granulocyte colony-stimulating factor (GCSF), and interleukin-1 alpha (IL-1α) exhibited a significant increase in expression on day 6. Conversely, tissue inhibitor of metalloproteinases 1 (TIMP-1), an inflammatory cytokine, showed high expression on day 2 but a significant decline on day 6 [Fig. 5(b)]. Differences in ${\mathrm{StO}}_{2}$ between the open wound and the unwounded skin on days 2, 6, and 12 are illustrated in Fig. 5(c), showing similar trends with the expression of proliferative markers (i.e., GCSF and IL-1α). Conversely, the inflammatory marker demonstrated an opposite trend to the change in tissue oxygenation. A high negative correlation coefficient of −0.73 was found between TIMP-1 and the differences in tissue oxygenation between open wounds and unwounded skin [Fig. 5(d)].

## 4 Discussion

In our study, the TOSD system demonstrated effectiveness in providing a noncontact and convenient method for detecting ${\mathrm{StO}}_{2}$, validated through the use of multiple imaging modalities. Specifically, three imaging systems were employed to monitor wound conditions in an animal model setting. This methodology allowed us to further demonstrate that the rate of wound closure increases during the proliferative phase, with TOSD and LSCI showing comparable changes in signals throughout the wound healing process. Notably, compared with intact skin, open wounds exhibited significantly higher signals as detected by both TOSD and LSCI. Furthermore, the levels of tissue oxygenation in wounded areas, as detected by TOSD, were found to correlate with the expression levels of angiogenesis markers, which were elevated during the proliferative phase of wound healing.

The wound area was quantified using digital photographs on each experimental day, and the corresponding wound closure rates were calculated. During the dynamic process of wound healing, the proliferative phase was observed to exhibit the most rapid wound closure. Previous studies have demonstrated the capability of NIR imaging in assessing wounds.^25^^,^^26^^,^^33^ In this study, the TOSD technique showed an advantage in correlating its signals with wound reduction by utilizing NIR technology. Signals captured by TOSD and LSCI were consistent with the trend of wound healing. Moreover, the relationship between ${\mathrm{StO}}_{2}$ and wound closure rate is stronger than that between blood flux and wound closure rate. Specifically, increases in ${\mathrm{StO}}_{2}$ correspond more closely to increases in wound closure rate compared with blood flux detected by LSCI. To further quantify the relationship between wound closure rate and ${\mathrm{StO}}_{2}$, we performed a linear regression analysis. The normalized regression coefficient (β) of ${\mathrm{StO}}_{2}$ and wound closure rate is 4.15, indicating that a 1% change in ${\mathrm{StO}}_{2}$ corresponds to ∼4% change in wound closure rate. The methodologies underlying TOSD and LSCI differ; LSCI operates based on the Doppler effect,^6^^–^^9^^,^^34^ whereas TOSD detects ${\mathrm{StO}}_{2}$ by directly irradiating ${\mathrm{HbO}}_{2}$ and Hb. LSCI calculates frequency shifts from scattered light upon moving objects, such as the blood, to deduce blood perfusion.^35^^,^^36^ Blood perfusion in the tissues is responsible for the delivery of oxygen and nutrients, as oxygen supply in the tissues is derived from oxygen diffusing through the microcirculation.^37^ Hence, blood perfusion in the tissue detected by LSCI can be used as an indirect indicator of tissue oxygenation.^38^ However, due to the variations in blood saturation throughout the body, particularly around wounds where oxygen consumption can be substantial, LSCI may not always provide sufficient information on tissue oxygenation. Another limitation of LSCI is the relatively small penetration depth of speckle images, which is limited to 1 mm.^9^^,^^39^ In contrast, TOSD, with a deeper penetration of ∼2 to 3 mm,^40^ is better suited for assessing deeper wounds and can assess more comprehensive ${\mathrm{StO}}_{2}$. TOSD exploits differences in the optical properties of cutaneous tissues, allowing for the estimation of tissue oxygenation parameters and subsequent calculation of ${\mathrm{StO}}_{2}$. Our findings demonstrated that wound oxygenation detection using TOSD better aligned with the proliferative phase compared with LSCI. Thus, our research suggests that TOSD offers enhanced capabilities for monitoring wound conditions and identifying the proliferative phase.

The proliferative phase represents a pivotal stage in the intricate cascade of events involved in wound healing. Characterized by the development of granulation tissue and the initiation of angiogenesis, this phase heavily relies on the activity of various cytokines, including proinflammatory cytokines and growth factors, which play indispensable roles in facilitating the healing process.^3^^,^^41^^–^^47^ Despite the recognized importance of angiogenesis markers in wound healing, previous research has not explored the correlation between these markers and NIR signals in wounds. In our study, biochemical analysis of wound exudate revealed a positive correlation between ${\mathrm{StO}}_{2}$ and proliferative cytokines, such as Granulocyte colony-stimulating factor (G-CSF)^41^^,^^45^ and Interleukin 1-alpha (IL-1α).^3^^,^^42^^,^^46^^,^^47^ Conversely, a significant negative correlation was observed between ${\mathrm{StO}}_{2}$ and the inflammatory cytokine tissue inhibitor, TIMP-1. These findings underscore the utility of the TOSD technique in discerning the proliferative phase of wound healing.

The efficacy of our TOSD system was validated through various methodologies. TOSD, utilizing low-power LEDs, has the potential for miniaturization, making it cost-effective and portable. Among noninvasive approaches, LSCI, HSI, MSI, and ultrasound have been used to assess tissue oxygenation and blood flow in wounds.^6^^,^^11^^,^^48^^–^^50^ However, LSCI is susceptible to ambient light, which limits its clinical application.^10^ HIS requires complex calculation processes and high computational power, which hinders widespread adoption and use.^13^^,^^14^ Ultrasound imaging, particularly Doppler ultrasound, can provide information on blood flow velocity and perfusion in superficial and deep tissues.^49^ However, it has limitations in operator dependence, susceptibility of artifacts, indirect measurement of tissue oxygenation, and the need for coupling gel application, which might affect the wound.^51^

Compared with these imaging techniques, TOSD offers several advantages. First, TOSD, as an MSI system based on NIR, has a relatively simple imaging technique that can directly measure tissue oxygenation using NIR wavelengths.^15^^–^^17^ Second, TOSD has relatively high resolution^52^^,^^53^ and is less sensitive to ambient light interference compared with LSCI.^10^ Third, TOSD has a lower cost and requires less computational power compared with HSI, making it more suitable for clinical settings.^15^

However, TOSD also has some limitations. Due to the limited penetration depth of NIR light, TOSD can only detect ${\mathrm{StO}}_{2}$ in superficial tissues (2- to 3-mm depth).^40^^,^^49^ This limitation may restrict its application in assessing deep tissue oxygenation (e.g., muscle layer). Also, in our current investigation, the precision of TOSD measurements could be further improved to ensure a more reliable assessment of tissue oxygenation.

Despite these limitations, TOSD remains a promising technique for the noninvasive assessment of tissue oxygenation in superficial wounds. Its relative simplicity, low cost, potential for miniaturization,^16^ and direct measurement of ${\mathrm{StO}}_{2}$ make it an attractive option for clinical wound assessment. The miniaturization potential of TOSD enables the development of compact and portable devices that can be easily used in various clinical settings, including remote locations, which is particularly useful for telemedicine applications. We expect TOSD to play a crucial role in aiding clinical decision-making regarding wound conditions and hold potential utility in telemedicine applications.

In our work, TOSD was exclusively utilized for monitoring open wounds created on the dorsum of mice. Future preclinical studies should involve experiments on larger animal models to further extend its translational applications. In addition, beyond incisional wounds, it is imperative to explore TOSD’s utility in assessing various types of wounds, such as venous ulcers, diabetic foot ulcers, and chronic wounds. Our ongoing research endeavors will focus on leveraging TOSD’s capability to gather critical information for predicting wound healing across diverse wound types. The application of our device extends to evaluating interventions aimed at facilitating wound healing, including novel wound dressings, topical growth factors, negative pressure wound therapy, and hyperbaric oxygen therapy, when integrated into wound management protocols.

## Supplementary Material



## Data Availability

The data that support the findings of this study are available from the corresponding author upon reasonable request.
