# Supplementary material for: Validation of multispectral imaging–based tissue oxygen saturation detecting system for wound healing recognition on open wounds
Source: J Biomed Opt. 2024 Aug 13;29(8):086004. doi: 10.1117/1.JBO.29.8.086004 (PMC11321076; doi:10.1117/1.JBO.29.8.086004)
Supplement: Supplementary file 1 [file JBO_029_086004_SD001.pdf]

Supplementary information

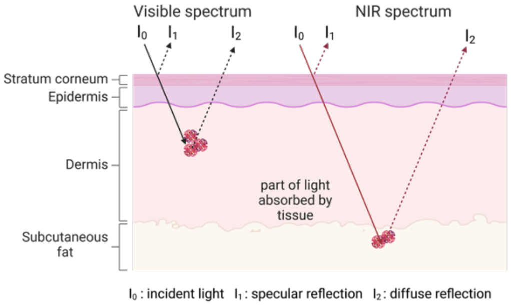

step1: $OD(\lambda) == ln\frac{I_0 - I_1}{I_c - I_1} = ln\frac{I_0 - R * I_0}{I_2 - R * I_0}$
$= (\varepsilon_{Hb}c_{Hb} + \varepsilon_{HbO_2}c_{HbO_2} + \varepsilon_{water}c_{water} + \varepsilon_{other}c_{other}) * d(\lambda)$

step2: *using* λ_1*=660nm*、λ_2*=880nm to solve* C_{Hb}, C_{HbO_2}

$$\frac{\varepsilon_{Hb}^{\lambda_1}c_{Hb} + \varepsilon_{HbO_2}^{\lambda_1}c_{HbO_2}}{\varepsilon_{Hb}^{\lambda_2}c_{Hb} + \varepsilon_{HbO_2}^{\lambda_2}c_{HbO_2}} = \frac{\frac{OD(\lambda_1)}{d(\lambda_1)} - \varepsilon_w^{\lambda_1}c_w - \varepsilon_o^{\lambda_1}c_o}{\frac{OD(\lambda_2)}{d(\lambda_2)} - \varepsilon_w^{\lambda_2}c_w - \varepsilon_o^{\lambda_2}c_o}$$

step3: *derive calculated value of* $StO_2 = \frac{C_{HbO_2}}{C_{Hb} + C_{HbO_2}}$

Supplementary Fig. S1. Signal detecting and calculation equation of TOSD. Based on the modified Beer-Lambert law, StO2 can be deduced following steps shown above. λ is the wavelength of the light source; $OD(\lambda)$ is the optical density and it equals the sum of the absorbances in cutaneous tissues; I_0 is the intensity of incident light; I_1 is the intensity of reflected light, and I_2 is the intensity of the scattered light. ε is the absorption coefficient of chromophores and *C* is the concentration of chromophores. $d(\lambda)$ is the maximum depth of penetration of the light (λ) in cutaneous tissues.

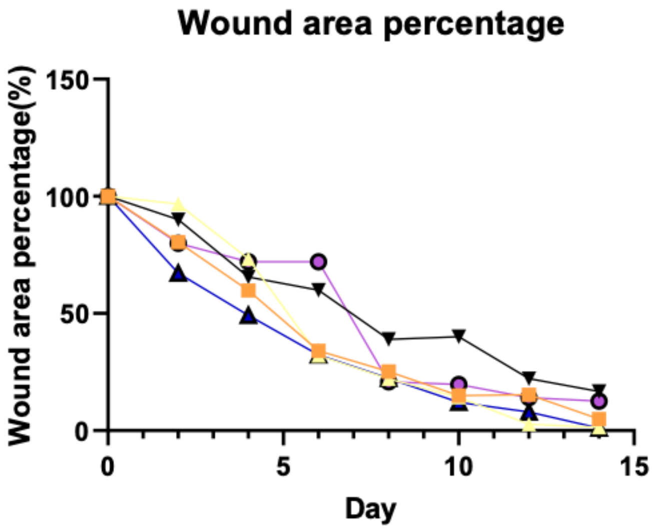

Supplementary Fig. S2. Raw data of wound area size percentage from Day0 to Day12.

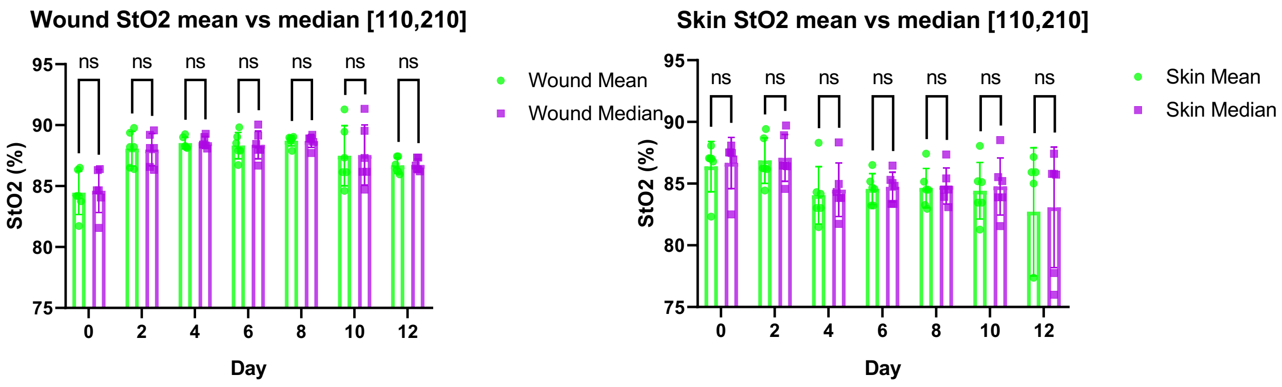

Supplementary Fig. S3. Comparison of average oxygenation presented by mean vs median in wound (left) or unwounded skin (right). Data are presented as mean value ± SD (n=5). ns indicates $p > 0.05$ by one-way ANOVA.
